# Enhanced bone tissue regeneration of a biomimetic cellular scaffold with co‐cultured MSCs‐derived osteogenic and angiogenic cells

**DOI:** 10.1111/cpr.12658

**Published:** 2019-07-11

**Authors:** Limei Li, Jidong Li, Qin Zou, Yi Zuo, Bin Cai, Yubao Li

**Affiliations:** ^1^ Research Center for Nano‐Biomaterials, Analytical & Testing Center Sichuan University Chengdu China; ^2^ Technology Transfer Center Kunming Medical University Kunming China

**Keywords:** angiogenic cells, biomimetic scaffold, bone tissue engineering, co‐culture, osteogenesis, stem cells

## Abstract

**Objectives:**

The bone tissue engineering primarily focuses on three‐dimensional co‐culture systems, which physical and biological properties resemble the cell matrix of actual tissues. The complex dialogue between bone‐forming and endothelial cells (ECs) in a tissue‐engineered construct will directly regulate angiogenesis and bone regeneration. The purpose of this study was to investigate whether co‐culture between osteogenic and angiogenic cells derived by bone mesenchymal stem cells (MSCs) could affect cell activities and new bone formation.

**Materials and methods:**

Mesenchymal stem cells were dually induced to differentiate into osteogenic cells (OMSCs) and ECs; both cell types were co‐cultured at different ratios to investigate their effects and underlying mechanisms through ELISA, RT‐qPCR and MTT assays. The selected cell mixture was transplanted onto a nano‐hydroxyapatite/polyurethane (n‐HA/PU) scaffold to form a cell‐scaffold construct that was implanted in the rat femoral condyles. Histology and micro‐CT were examined for further verification.

**Results:**

ELISA and gene expression studies revealed that co‐cultured OMSCs/ECs (0.5/1.5) significantly elevated the transcription levels of osteogenic genes such as ALP, Col‐I and OCN, as well as transcription factors Msx2, Runx2 and Osterix; it also upregulated angiogenic factors of vascular endothelial growth factor (VEGF) and CD31 when compared with cells cultured alone or in other ratios. The optimized OMSCs/ECs group had more abundant calcium phosphate crystal deposition, further facilitated their bone formation in vivo.

**Conclusions:**

The OMSCs/ECs‐scaffold constructs at an optimal cell ratio (0.5/1.5) achieved enhanced osteogenic and angiogenic factor expression and biomineralization, which resulted in more effective bone formation.

## Graphical abstract

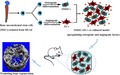

## 1 INTRODUCTION

Bone fracture healing is a complex process mediated by multiple factors; many cell types are involved in the formation, repair and remodelling of bone.^1^ Over the past decade, a biomimetic scaffold seeded with a single cell type—such as osteoblasts, bone marrow stromal cells or mesenchymal stem cells expanded in vitro—in a state that guarantees their differentiation into functional bone matrix‐producing cells has been considered as an alternative to bone grafting.^2^ Following the recognition of the limits associated with mimicking complex biological environments when introducing single‐cell phenotypes, the co‐culture of two or more types of cells in vitro and in vivo is now being granted more attention due to their ability to more closely model natural bone regeneration. This provides additional insight into that cell‐cell interactions may improve the efficiency of current bone tissue engineering.^3^, ^4^

Cell‐cell communication between diverse cell types is vital to the tissue healing process.^5^, ^6^ Cells co‐cultured with other cell types can produce bioactive factors that allow different crosstalk schemes between cells, promoting endocrine, paracrine, autocrine, and electric signalling routes and direct effects that are dependent on cell contact. Several studies have shown synergistic effects in response to the use of co‐culture systems, which have the ability to induce stem cell differentiation.^7^, ^8^ The previous studies suggested that the synergistic interplay between osteogenesis and angiogenesis plays a pivotal role in the bone regeneration process,^9^, ^10^ while rapid revascularization is crucial for transplanted cell survival and new bone formation. Because bone is a calcified and peripherally vascularized tissue consisting of various cell types, including osteogenic cells and endothelial cells, co‐culture of cells with osteogenic and angiogenic potential draw much attention in bone tissue engineering.^5^ Herzog et al found that the co‐culture of primary osteoblasts and the outgrowth of endothelial cells (ECs) positively influenced vessel formation and bone repair, which was associated with rising levels of growth factors and proteins of different origins.^11^ Osteoblasts produce angiogenic factors, such as vascular endothelial growth factor (VEGF) and matrix components, which are important in vessel component differentiation; in turn, these factors stimulate ECs to produce osteogenic factors, such as BMP‐2.^12^, ^13^ The association of these two essential cell types in a biomaterial can provide a live bone graft that can be used to repair bone defects,^14^ which may be beneficial for rebuilding the vascular network within tissue‐engineering constructs and subsequently promoting bone tissue regeneration.

In addition to the selection of co‐cultured cell types, the ratio of the different cell types in the co‐culture system can also influence cell characteristics, survival and behaviours. Therefore, the proper ratio of co‐cultured cells may be important to guarantee an excellent bone tissue‐engineering construct. However, in view of the available literature, few systematic studies assessing optimal cell ratios between ECs and tissue‐specific cells have been reported. In most studies, researchers selected a 1:1 cell ratio^15^, ^16^; however, this may be a matter of keeping things simple, rather than utilizing the full potential of co‐cultures.^17^, ^18^ An early study by Kim et al reported that the optimal ratio (0.5/1) of two different cell types, adipose‐derived stromal cells (ASCs) and bone marrow stromal cells, promoted osteogenic differentiation and osteogenesis in a co‐culture model.^19^ The effect of the co‐cultured cells at different ratios was also investigated by Ma et al using human umbilical ECs and human marrow stromal cells^20^; however, the optimal ratio (1:1) of co‐cultured cells remained poorly understood and required more systematic investigations. To optimize the co‐cultured cell ratio, the various tests should not only investigate the proliferation or viability of cells, but also assess the impact of this ratio on gene expression, the related signal‐transduction pathway and the desired phenotypic expression within the co‐culture system.

Mesenchymal stem cells, which are primarily present in bone marrow, are multipotent stem cells that can differentiate into target cells such as osteoblasts,^21^ chondrocytes^22^ and endothelial cells^23^ under specific conditions. MSCs have been extensively investigated and were shown to be the most suitable cell source for bone tissue engineering due to their excellent osteogenic potential; furthermore, researchers also revealed that MSCs promote angiogenesis through proteolytic mechanisms.^24^ Considering the lower osteogenic potential of MSCs compared with osteogenic‐induced differentiated MSCs (OMSCs),^25^ we hypothesize that the co‐culture of MSCs‐derived osteogenic and angiogenic cells at an optimal ratio may be a promising strategy for vascularized bone tissue regeneration. The purpose of this study was to investigate whether the co‐culture of MSCs‐derived ECs to OMSCs, as well as cell ratio, affected cell activities and new bone formation. Thus, we first induced the osteogenic and angiogenic differentiation of MSCs into OMSCs and ECs, respectively. Subsequently, the optimal ratio of OMSCs/ECs in the co‐culture system was determined by exploring the level of cell crosstalk based on the functional markers of osteogenic and angiogenic expression. After screening for the optimal ratio of OMSCs/ECs using in vitro experiments, the selected co‐cultures were transplanted onto a biocompatible and bioactive n‐HA/PU composite scaffold to form a cellular bone graft.^26^ A condylar femur defect model in rat was used to demonstrate the effect of new bone formation.

## 2 MATERIALS AND METHODS

### 2.1 Cell isolation and cultivation

**Figure 1:**
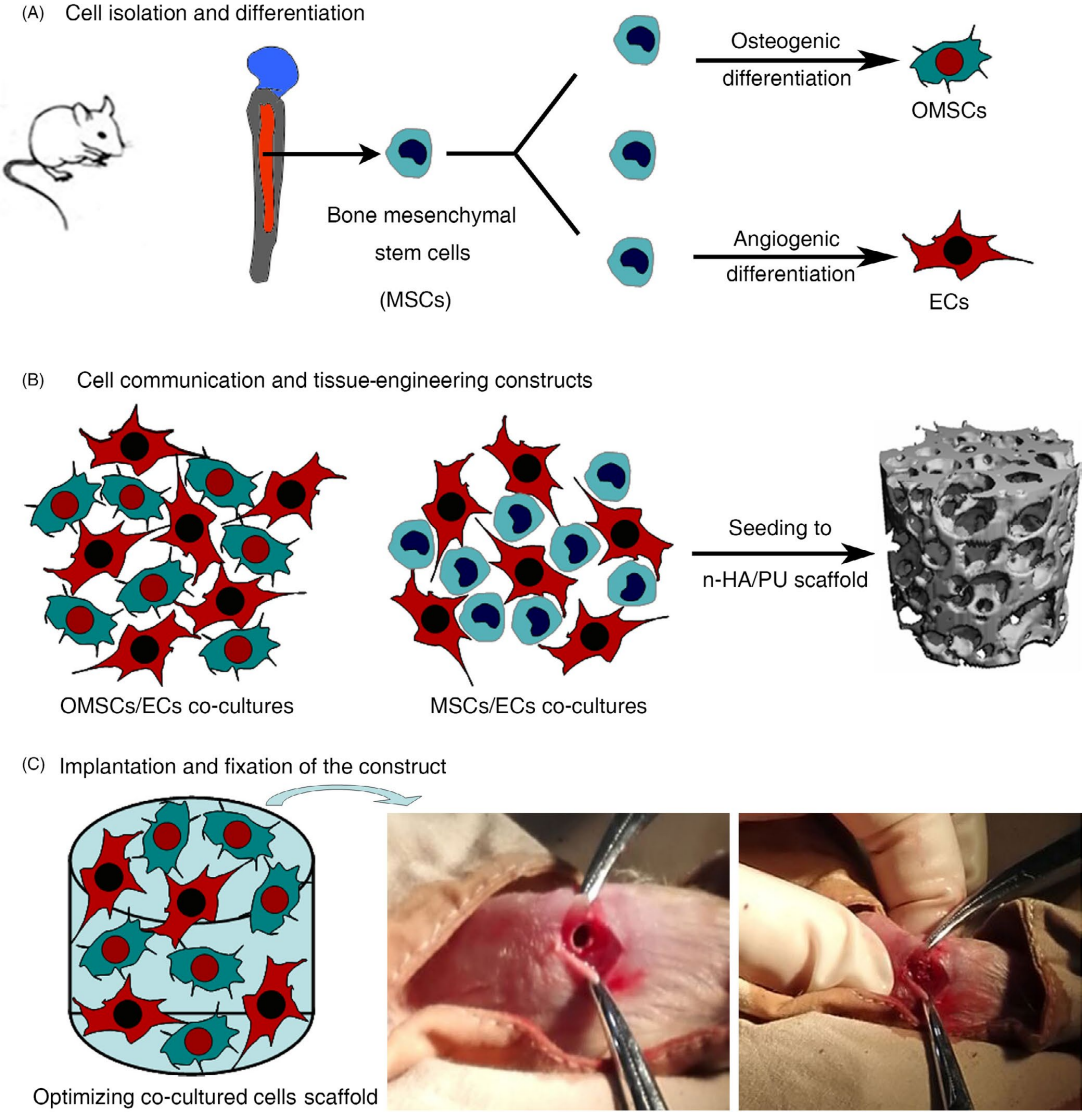
Schematic representation of the in vitro and in vivo experimental procedure. A, MSCs isolation and dual induced differentiation. B, Co‐culture model systems used for the analysis of cell‐to‐cell interactions and their mixtures at optimal ratios co‐cultured with the n‐HA/PU scaffold. C, In vivo experimental procedure

Sprague Dawley (SD) rats of about 100 g in weight were used as donors of femurs and tibiae for bone marrow harvesting and primary MSCs isolation, according to an established procedure (Figure 1A). Briefly, bone marrow was flushed out using α‐minimum essential medium (α‐MEM) supplemented with 1% antibiotic/antimycotic and 20% foetal bovine serum. Cells were plated on a culture flask, changing α‐MEM every 3 days. After 1 week of incubation, MSCs were regularly subcultured and the fourth passage cells were used for experiments.

#### 2.1.1 Osteogenic‐differentiated MSCs

For osteogenic induction, the MSCs were cultured for 14 days in osteogenic medium (OM: α‐MEM medium containing 1% antibiotic/antimycotic, 10% foetal calf serum, 50 mg/L ascorbate, 10 mmol/L glycerophosphate and 0.1 μmol/L dexamethasone). The OMSCs phenotype was identified by alkaline phosphatase (ALP) and Alizarin red S staining.

#### 2.1.2 Vascular endothelial‐differentiated MSCs

For angiogenic induction, the MSCs were cultured for 14 days in angiogenic medium (α‐MEM: α‐MEM medium containing 1% antibiotic/antimycotic, 20% calf serum, 10 μg/mL VEGF and 2 μg/mL basic fibroblast growth factor [b‐FGF]). Then, the ECs phenotype was confirmed by VEGF and CD31 staining.

### 2.2 In vitro mixed co‐culture of OMSCs/ECs

In vitro mixed co‐cultures of OMSCs and ECs at the fourth passage were carried out in OM shown in Table 1. As controls, the monoculture of OMSCs and ECs was performed in the same cell numbers in OM and α‐MEM separately.

**Table 1:** Groups of different co‐cultured cell mixtures

Total cell numbers	OMSCs/ECs
2.0 × 10^5^	2.0 × 10^5^/‐‐
	1.5 × 10^5^/0.5×10^5^
	1.0 × 10^5^/1.0 × 10^5^
	0.5 × 10^5^/1.5 × 10^5^
	‐‐/2.0 × 10^5^

#### 2.2.1 Proliferation assay

The proliferation of different cells types for 4, 7 and 14 days of monoculture or co‐culture was evaluated by MTT (3‐[4,5‐dimethylthiazol‐2‐yl]‐2,5‐diphenyl‐2H‐tetrazolium bromide; Amresco, USA) assay with a multilabel counter (Wallac Victor3 1420; PerkinElmer Co) at 490 nm.

#### 2.2.2 ELISA assay

From the co‐culture settings, the medium was taken from the culture flask after 3 days of in vitro culture and assayed to measure the level of ALP, osteocalcin (OCN) and VEGF. ALP and OCN assays (Thermo Fisher Scientific, Waltham) were performed to detect early osteogenic cell differentiation. A VEGF ELISA kit (Thermo Fisher Scientific) was used to quantify VEGF, according to the manufacturer's instructions.

#### 2.2.3 Reverse transcription and quantitative polymerase chain reaction

The osteogenic and angiogenic differentiation of co‐cultured cells was further assessed by real‐time quantitative polymerase chain reaction (RT‐qPCR) to measure the mRNA expression of ALP, Msx2, Runx2, Osterix, Col‐I, OCN, VEGF and CD31. Cells above a specified density were seeded in a culture flask and incubated in the relevant medium for 14 days. Once the cells were set, the total RNA was isolated using Trizol reagent (Thermo Fisher Scientific) according to the manufacturer's instructions. A total of 1.5 μg RNA was reverse‐transcribed with 0.5 μL of oligo (dT). The RT‐qPCR was performed in a 20‐μL standard reaction: 10 μl 2× SYBR Green PCR Master Mix; 0.5 μL forward primer and 0.5 μL reverse primer; and 5 μL diluted cDNA. The cycle threshold (CT) values were used to calculate the relative fold‐change based on the value of control sample (2^−ΔΔCT^ method). β‐actin served as an internal normalized reference. Three parallel samples were used for this test. The primer pairs used are listed in Table S1.

### 2.3 Morphology and mineralization of MSCs, OMSCs, ECs and co‐cultured cells on the n‐HA/PU scaffolds

The n‐HA/PU scaffold was prepared according to our previous report^26^ and cut into square samples (10 × 10 × 2 mm^3^). After an ultrasonic rinse in distilled water and sterilization with an autoclave, the samples were seeded with cells statically. The selected ratio of the co‐cultures with the scaffold is presented in Table 2; constructs of OMSCs/ECs were cultured in OM, while the same ratio of MSCs/ECs was in α‐MEM medium in 24‐well plates as control in a humidified incubator (37°C, 5% CO_2_).

**Table 2:** Constructs of optimal co‐cultured cell mixtures seeded on n‐HA/PU scaffolds

OMSCs/ECs	MSCs/ECs
2.0 × 10^4^/‐‐	2.0 × 10^4^/‐‐
0.5 × 10^4^/1.5 × 10^4^	0.5 × 10^4^/1.5 × 10^4^
‐‐/2.0 × 10^4^	

The morphology and spreading of cells growing on the scaffolds were observed by scanning electron microscopy (SEM; JSM‐6510LV, JEOL) and fluorescence microscopy (TE 2000‐U; Nikon Eclipse). Before SEM observation, the samples were rinsed with phosphate‐buffered saline (PBS), fixed with 2.5 vol% glutaraldehyde, dehydrated through graded ethanol and dried using the CO_2 _critical point‐drying method. For fluorescence observations, the cells were labelled with the live/dead reagent (Live/Dead Viability/Cytotoxicity Kit; Thermo Fisher Scientific).

### 2.4 Rat condyle: femur defect repair

#### 2.4.1 Construction of n‐HA/PU‐seeded cells

Scaffolds were cut into cylinders (diameter: 3 mm; thickness: 3 mm) using a trephine bur. Following ultrasonic rinse in distilled water and sterilization with an autoclave, the scaffolds were incubated overnight in fresh α‐MEM and then co‐cultured with cells. For the co‐cultured series design, as stated in Section 2.3, the cell suspensions were statically seeded on the scaffold (where the scaffold was without cells as control) and cultured for 14 days in a CO_2_ incubator at 37°C to obtain the cellular constructs (Figure 1B).

#### 2.4.2 Construct implantation

A total of 20 SD rats with a weight of about 200 g were used in accordance with the protocol approved by the Ethics Committee of West China Hospital of Sichuan University in compliance with all regulatory guidelines. The anaesthesia (chloral hydrate, 1.5 mL/kg) for all animals was administered intraperitoneally. To reduce the peri‐operative infection risk, the rats received antibiotic prophylaxis. Following exposure of the distal femoral condyle, a defect 3 mm in diameter and 3 mm in depth was created using a trephine bur under continuous saline buffer irrigation. Thereafter, the cellular constructs were implanted into the condylar femur defects of a rat (Figure 1C). For the blank control group, the same defects were created without any treatment. The implants were harvested for analysis after 4 and 8 weeks and fixed in 4% phosphate‐buffered paraformaldehyde solution.

#### 2.4.3 Microcomputed tomography scanning

The implants (n = 3) were analysed using a micro‐CT system (*μ*CT80 scanner; Scanco Medical AG). The parameters were set at a resolution of 19.5 μm, along with an energy source of 70 kVp and a 114 μA current, and a 3D Gaussian filter was constrained at *σ* = 1.4 and support = 2 for partial suppression of the noise in the test volumes. The full region of each implant was scanned and, on average, consisted of 154 slices. The three‐dimensional bone was reconstructed and analysed using a threshold of 226‐1000. The obtained micro‐CT images were analysed for the quantitative evaluation of osteogenesis on and in the porous scaffold, employing the Direct Method software attached to the micro‐CT to derive the bone volume to tissue volume (BV/TV), trabecular thickness (Tb.Th) and trabecular separation (Tb.Sp).

#### 2.4.4 Histological evaluation

The samples were decalcified and then dehydrated through gradient ethanol, cleaned in xylene and embedded with paraffin wax. Finally, the samples were cut into sections (5 μm in thickness) along the sagittal plane, stained with haematoxylin and eosin (HE) staining and observed under optical microscopy.

### 2.5 Statistical analysis

Quantitative data were presented as the mean ± standard deviation (SD). Statistical analysis was carried out using one‐way analysis of variance (ANOVA) with a Tukey test. Differences were considered to be statistically significant when *P* < 0.05.

## 3 RESULTS

### 3.1 Effects of ECs in mixed co‐culture with OMSCs

According to the existing and well‐known methods used to induce stem cell differentiation, we separately induced MSCs differentiation into OMSCs^25^ and ECs^27^ under specific conditions. The OM‐induced osteoblastic phenotype of OMSCs was confirmed by ALP and Alizarin red S staining. After 14 days of osteogenic induction, the cells showed positive staining for ALP, and the mineralized nodules were appeared in Alizarin red S staining at 28 days of culture (Figure S1). The successful induction of MSCs differentiation into ECs was also identified by the positive staining of CD31 and VEGF markers (Figure S2). Thus, we successfully obtained two desired cell phenotypes derived from same MSCs source by different induction conditions, which could be used for the subsequent experiments.

**Figure 2:**
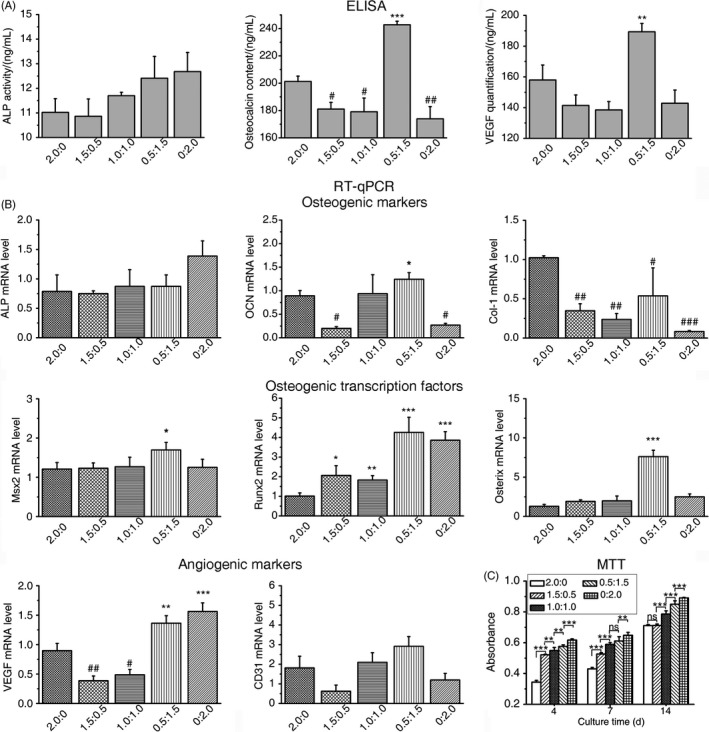
Effects of ECs in the mixed co‐culture with fixed numbers of total ECs and OMSCs. A, ELISA for ALP, OCN and VEGF markers after 3 d. B, RT‐qPCR for osteogenic (ALP, OCN, Col‐I, Msx2, Runx2 and Osterix) and angiogenic (CD31 and VEGF) markers after 14 d. The number of total cells was fixed at 2.0 × 10^5^ and “2.0:0” indicates that 2.0 × 10^5^ OMSCs were co‐cultured with 0 ECs. C, Effects of ECs in a mixed co‐culture with OMSCs on cell proliferation in the OM on days 4, 7 and 14. The total cells proliferated with increased ECs numbers. The bar represents the mean ± SD. N = 3; *significantly greater than OMSCs mono‐culture; ^#^significantly lower than OMSCs monoculture

To determine the interaction between OMSCs and ECs, the optimal cell ratio between these two cell types and their effect on osteogenesis and angiogenesis, the co‐cultures were utilized in fixed numbers of total cells (2.0 × 10^5^) with variable ratios of ECs to OMSCs. It is apparent that the group with a ratio of 0.5/1.5 had the highest OCN and VEGF content, as determined by the ELISA data (Figure 2A). Moreover, VEGF amount in this group is even higher than the ECs monoculture group. The effects of osteogenic‐induced OMSCs on the gene expression of co‐cultured OMSCs/ECs were also mirrored by RT‐qPCR. It was found that the level of osteogenic genes (OCN, Msx2, Runx2, Osterix) expression was relatively higher at the ratio of 0.5/1.5 (Figure 2B). When the vascular genes (CD31 and VEGF) were detected, the level varied with different ratios; however, the highest level was achieved with an OMSCs/ECs ratio of 0.5/1.5. MTT assays demonstrated that the total cells proliferated with increased ECs numbers and culture time. Overall, the co‐cultured cells of OMSCs/ECs at ratio of 0.5/1.5 were advantageous for osteogenic and vascular expression when compared with the other ratio.

### 3.2 Calcium phosphate precipitation

**Figure 3:**
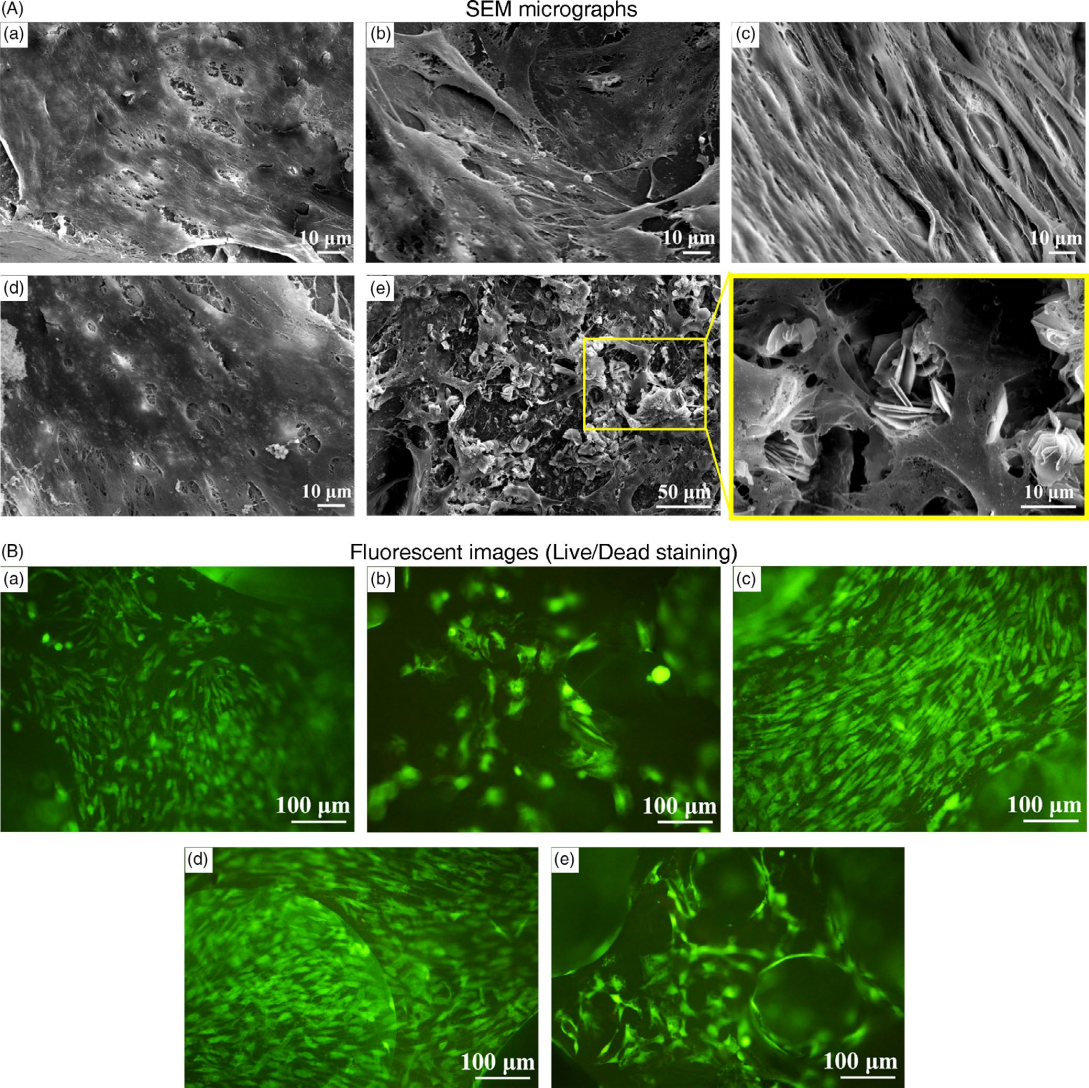
A, SEM micrographs and (B) fluorescent images (Live/Dead staining) of cells on the surface of the n‐HA/PU scaffold for 7 d: (a) MSCs group, (b) OMSCs group, (c) ECs group, (d) MSCs/ECs (0.5/1.5) group and (e) OMSCs/ECs (0.5/1.5) group. Effects of ECs in mixed co‐culture with OMSCs on cell proliferation and mineralization in OM medium. The flower‐like apatites are made up of flake apatite and inserted into the cell layers. One cell type mono‐culture and MSCs/ECs (0.5/1.5) as the control, MSCs, ECs and MSCs/ECs cultured in α‐MEM, OMSCs cultured in OM medium

After confirming that a number of ECs had a positive effect on the osteogenic differentiation of OMSCs when cultured in a mixed state, we then tried to investigate the enhancement of mineralization in the co‐culture systems in vitro. With respect to the cells that were cultured on the n‐HA/PU scaffold in vitro, it was revealed that those cells that adhered to the scaffold exhibited diverse mineralization and different viability upon SEM and fluorescence staining (Figure 3). The cells that attached to the surface of the porous scaffold presented with abundant pseudopodia and cytoplasmic extensions. It appears that osteogenic‐induced group (OMSCs/ECs) in the OM promoted mineralization (Figure 3A (e)), which yielded an abundance of calcium phosphate crystals, as demonstrated by energy‐dispersive X‐ray spectroscopy (EDS) analysis (Figure S3). The flower‐like crystallites were stacked regularly from lamellar crystal and were mainly located at the microfilament of cells.

As controls, four groups of OMSCs, MSCs, ECs and MSCs/ECs that were, respectively, seeded on the scaffolds were also investigated (Figure 3(a‐d)). Although OMSCs seeded scaffold was cultured in OM medium, only few mineral particles were found on or in the scaffold. The pictures also confirmed that the cell densities of osteogenic‐induced group (OMSCs/ECs, OMSCs) were lower than that in the non‐induced groups (MSCs, ECs, MSCs/ECs). It is known that differentiated cells (OMSCs) proliferate less than undifferentiated mates (MSCs), which may be due to an inhibitory effect that occurred by inducing factors. Furthermore, abundant ECs formed an arrangement on the scaffold.

### 3.3 In vivo effects of cellular constructs on bone regeneration

To evaluate the different cell mixture types on angiogenesis and osteogenesis in vivo, the cellular constructs were implanted in the bilateral femoral condyles of rats. There was no evident inflammation found, and the new bone tissue formation that occurred during defect healing was grossly evaluated using micro‐CT and microscopically assessed using HE staining at different time points.

**Figure 4:**
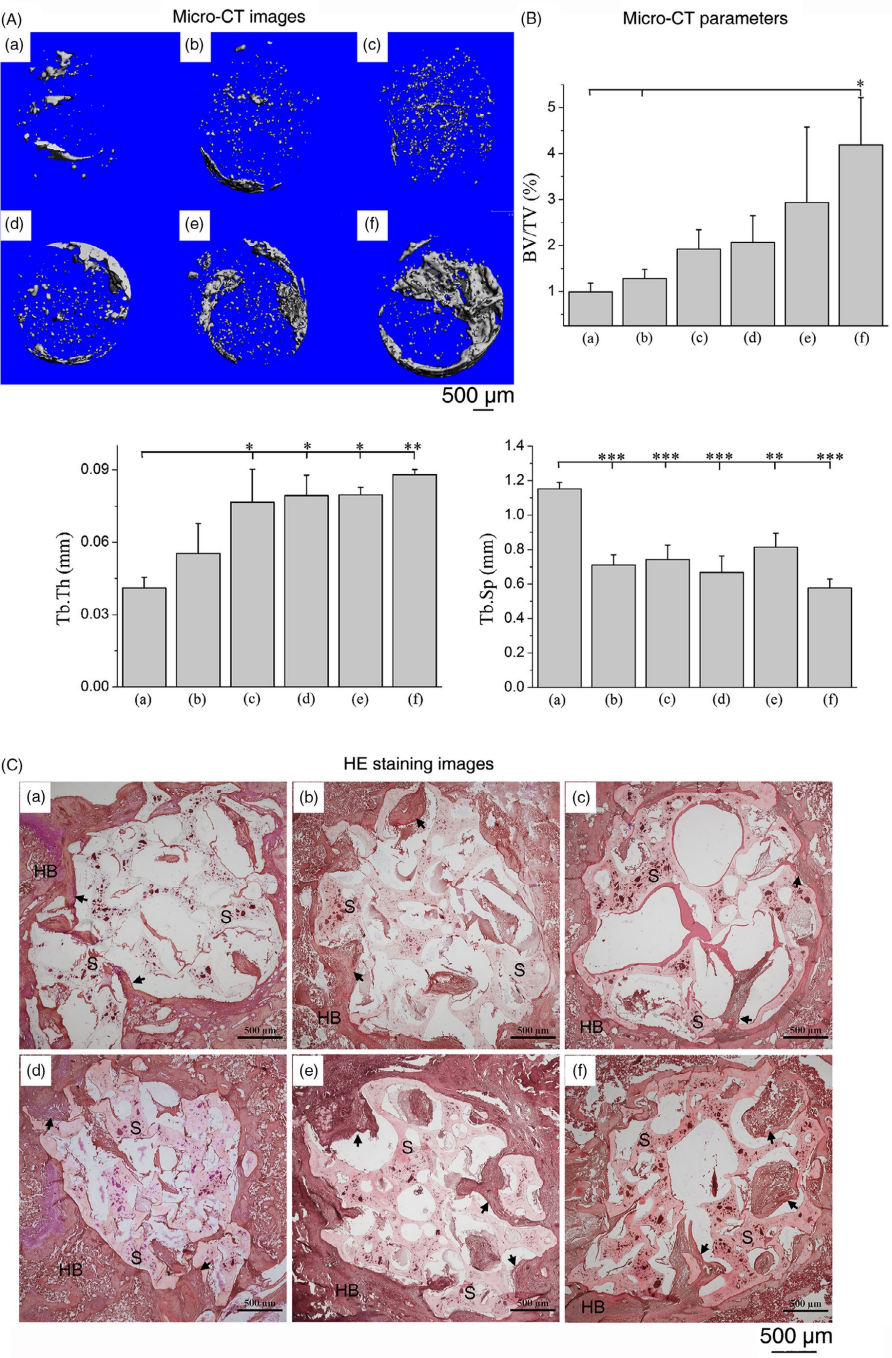
A, Micro‐CT images of the regenerated bone tissue and (B) relevant bone parameters of BV/TV (bone tissue volume/total volume), Tb.Th (trabecular thickness) and Tb.Sp (trabecular separation). C, Histological evaluation (HE staining) of new bone formation within the pore at 4 wk: (a) pure scaffold, (b) MSCs scaffold, (c) OMSCs scaffold, (d) ECs scaffold, (e) MSCs/ECs (0.5/1.5) scaffold and (f) OMSCs/ECs (0.5/1.5) scaffold. HB—host bone; S—scaffold; black arrows—new bone. *P < 0.05; **P < 0.01; ***P < 0.001

**Figure 5:**
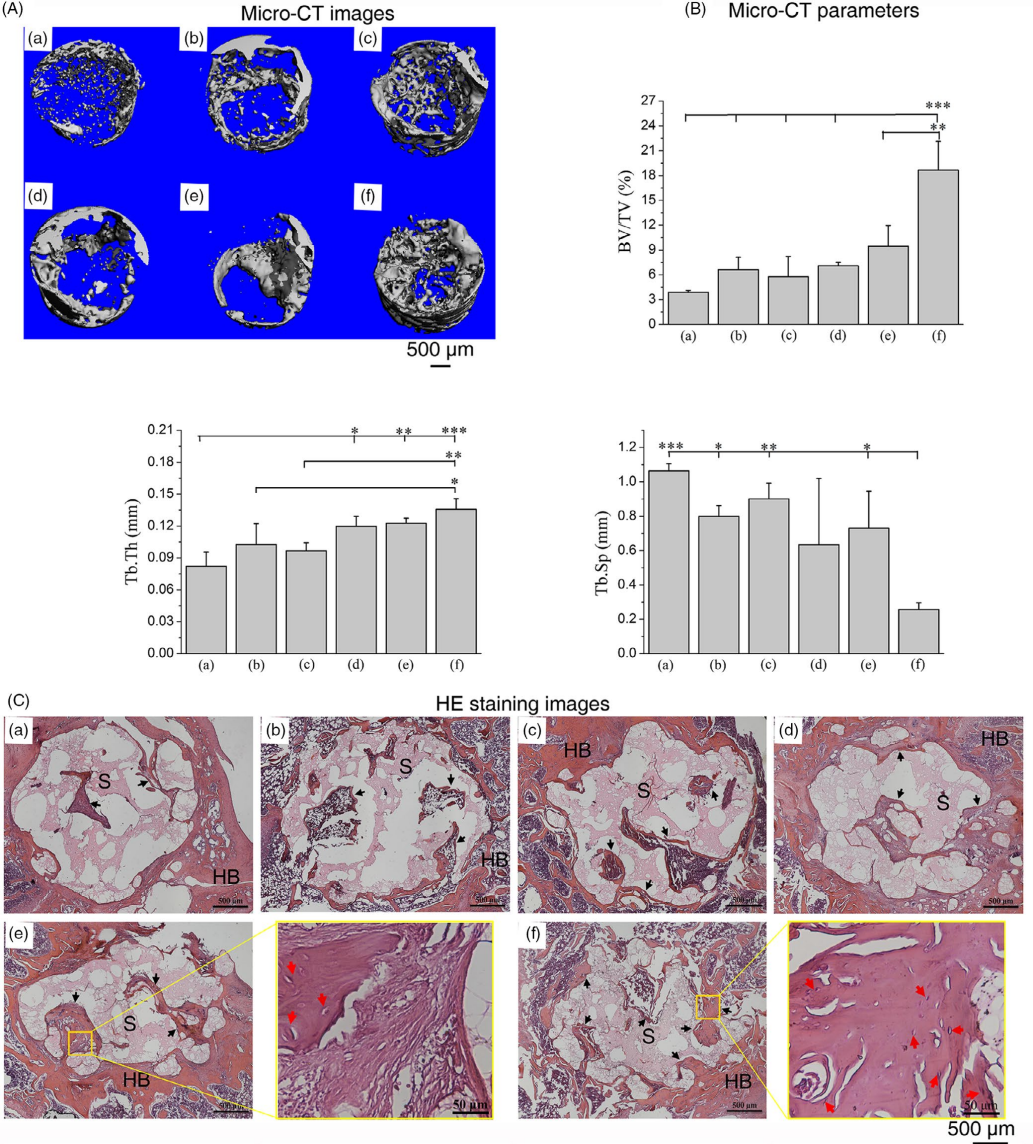
A, Micro‐CT images of the regenerated bone tissue and (B) relevant bone parameters of BV/TV (bone tissue volume/total volume), Tb.Th (trabecular thickness) and Tb.Sp (trabecular separation). C, Histological evaluation (HE staining) of new bone formation within the pore at 8 wk: (a) pure scaffold, (b) MSCs scaffold, (c) OMSCs scaffold, (d) ECs scaffold, (e) MSCs/ECs (0.5/1.5) scaffold and (f) OMSCs/ECs (0.5/1.5) scaffold. HB—host bone; S—scaffold; black arrows—new bone; red arrows—capillary vessels. *P < 0.05; **P < 0.01; ***P < 0.001

From the micro‐CT images at 4 weeks post‐implantation (Figure 4A), the bone tissue at the defect site appeared to be cancellous and can be primarily observed at the periphery of the scaffold. Treatment with different cell mixtures (Figure 4A [e, f]) significantly increased the mineralized bony area when compared to the single cell‐scaffold groups (Figure 4A [b–d]), while the bone repair capability of the pure scaffold (Figure 4A [a]) was weakest. It is important to note that the implants with OMSCs/ECs showed greater new bone formation and faster healing rates than did the MSCs/ECs group. At 8 weeks (Figure 5A), new bone with a higher density was formed around the scaffold, and the bone matrix and trabecula also grew into the porous structure, which revealed a similar but ascending trend when compared with the findings associated with the various samples at 4 weeks. The micro‐CT images at 8 weeks also revealed that the defects treated with OMSCs/ECs mixtures had a notably greater area of regenerated bone than those treated with MSCs/ECs mixtures. These trends were also confirmed by the increase in new bone volume density (BV/TV) and trabecular thickness (Tb.Th), as well as by the decline in the trabecular separation (Tb.Sp) data presented in Figures 4B and 5B.

The results of the micro‐CT analysis of the new bone formation that occurred at the defects were further supported by histological analysis (Figures 4C and 5C). All groups performed well in terms of the integrated peripheral surface of the scaffold and bone tissue (Figure 4C). The group with the pure scaffold did not form mature bone at 4 weeks, and it exhibited little bone tissue generation at 8 weeks. As for the groups with a cell‐loaded scaffold, a few mineralized matrix areas were generated around the materials at 4 weeks and grew into the pore of the scaffold at 8 weeks. The group of MSCs/ECs exhibited small areas of trabecular bone at 4 weeks of implantation, which became thicker in the newly generated bone region at 8 weeks. The group of OMSCs/ECs presented a mineralized matrix at 4 weeks, followed by the development of a mature trabecular bone meshwork. These results suggested that the OMSCs/ECs mixtures co‐cultured had a greater effect on bone formation and integrity when compared with the MSCs/ECs mixtures, which promoted a rapid bone‐healing process. In addition, the co‐cultured cells groups showed more active vascularization than the groups featuring either the scaffold alone or the scaffold with monoculture. Although capillary vessels (red arrow) in the groups of MSCs/ECs occurred at some sites in the trabecular bone, the numbers of capillaries were significantly less than those of the OMSCs/ECs group at 8 weeks (Figure 5C).

## 4 DISCUSSION

Clinical situations that require cell transplantation for bone regeneration are usually accompanied by poor vascular supply. However, rapid revascularization is a crucial factor for maintaining the survival of transplanted cells and for ensuring new bone formation. Therefore, the improved scaffold functionality achieved by pre‐seeding osteoblasts and endothelial cells is considered to be an effective approach for the survival of implanted cells and vascular bone formation.^28^ Co‐culture provides a powerful tool to promote cell differentiation due to cellular interactions with other cell types, such as trophic effects and cytokines, and it induces new bone tissue regeneration. The combination of different cell types on a biomimetic scaffold is also an approach that can be employed to obtain a closer representation of the complex crosstalk that occurs in natural tissues.^29^, ^30^ Recently, co‐cultured endothelial cells and stem cells displayed significantly enhanced expression levels of key osteogenic and vascular markers.^31^

An investigation of the secreted factors that are elevated during co‐culture can provide cues on the mechanisms underlying this process. Understanding the molecular processes that occur in a co‐culture may provide a means through which to mimic the cell environment. Despite the achievements made in the successful application of co‐cultured cells when treating bone defects in animals, the effective crosstalk, secretion of cytokines and co‐culture signals at optimal ratios required further elucidation. While the interaction between MSCs or osteoblasts and vascular ECs has been studied,^32^ the effects of the co‐culture of MSCs‐derived osteogenic and angiogenic cells on angiogenesis and osteogenesis have not been extensively investigated. In this study, we focused our attention on the interaction and optimal ratio of the co‐cultured MSCs‐derived OMSCs and ECs cells in in vitro evaluations and further confirmed their positive effects in vivo when seeding co‐cultures on n‐HA/PU scaffold.

This study obtained OMSCs by inducing the osteogenic differentiation of MSCs seeded in an osteogenic induction medium, and the cells were further co‐cultured with MSCs‐derived ECs at a certain ratio. Our results showed that osteogenic and angiogenic effects were the greatest at a certain optimal ratio of 0.5/1.5 (OMSCs/ECs) for the fixed total number series. When compared to the MSCs/ECs or mono‐culture groups, it was found that the OMSCs/ECs group at optimal ratio could promote rich mineralization on the surface of the cellular scaffold. The results can be attributed to the cell‐to‐cell contact in—and paracrine mechanism of—the co‐culture system. Osteogenic markers such as ALP, Col‐I and OCN, as well as the key transcription factors of Msx2, Runx2 and Osterix, were upregulated in the OMSCs/ECs co‐culture system. The BMP‐2 pathway is known to independently upregulate Osterix expression through two distinct transcription factors: Runx2 and Msx2.^33^ It can be speculated that in our OMSCs/ECs co‐culture, both Msx2/Osterix and Runx2/Osterix played positive roles during osteoblastogenesis, which were activated by the BMP‐2 signalling pathway; the upregulated transcription of osteogenic genes such as ALP, OCN and Col‐I; and the stimulated deposition of calcium crystals. Moreover, the expression of endothelial markers—that is CD31 and VEGF—also markedly increased with the optimal ratio of co‐cultures. VEGF and BMP‐2 might cooperate through a paracrine pathway, subsequently regulating osteogenesis and angiogenesis. Deckers et al reported that the BMP‐2 pathway elevated the expression of VEGF in ECs.^34^ Via their autonomous paracrine roles on angiogenesis and osteogenesis, cell signals from both cell types could diffuse in the extracellular environment and interact with the target cells through specific receptors.^5^

Apart from the positive effect of the co‐culture ratios on osteogenic outcomes, the osteogenic medium is required to differentiate the stem cells, thereby inducing mineralization.^35^ The addition of supplements to the medium is another issue that must be considered, as this markedly accelerates the osteogenic process. The endogenous factors secreted by the cells in the microenvironment may contribute to, or inhibit, the typical effects of supplements in the medium.^4^ Our findings showed that OMSCs/ECs cultured in the induction medium caused markedly mineralization at 7 days based on SEM images (Figure 3A(e)). More importantly, an osteogenic medium can maintain the strong osteogenic potential of OMSCs in co‐cultures, as well as the robustly active calcium deposition when compared with MSCs. For OMSCs group, we could also see few mineral particles on the scaffold surface, and it can be speculated more mineral formation with the extension of time. The results from Chan et al showed visible granules and flakes deposits on the biomaterial for 21 days.^36^ In addition, the higher VEGF amount and CD31 expression in OMSCs/ECs group (Figure 2) indicated that ECs in OM may keep their function appropriately.

Bone‐forming cells and endothelial cells engage in multiple interactions during bone formation. The positive effect of biomimetic co‐cultured cellular constructs on in vivo bone regeneration was evaluated by a condyle defect model in rat. A functional scaffold (ie supports growth and differentiation of the seeded cells) can trigger the cells networking and their interaction with the surrounding biomaterials.^37^ In this study, we selected biomimetic n‐HA/PU composite scaffold as a substrate, which has been proved to provide a support structure, enhance cell engraftment and survival, and further produce strong vitality in bone regeneration and reconstruction.^26^ We found that mixtures of OMSCs/ECs were more effective in inducing bone repair, and it facilitated better restoration of osseous structures than mixtures of MSCs/ECs or the application of one cell type alone, while the control (without constructs) presented the weakest bone repair capabilities, indicating prior mineralization in vitro (Figure 3) that coincided with more efficient bone tissue formation in vivo (Figures 4 and 5 [f]). This enhancement is the result of synergistic communication and mutual promotion between the two cell types—namely osteogenic‐angiogenic coupling. ECs, which can secret osteogenic factors in a co‐culture model, may involve in mediation stem cells towards the osteoblastic phenotype. In parallel, bone‐forming cells secrete angiogenic (such as VEGF) and osteogenic (such as BMP‐2) factors, which mediate the crosstalk between bone‐forming cells and ECs.^38^, ^39^ In view of the complicated interactions between the two cell types during the co‐culture period, the cell ratio at the sample collection timepoint might be quite different from the initially documented status. Although not determined in the present study, related results have been reported by Fuchs et al^40^ ECs displayed high proliferation and survival potential, as evidenced by MTT outcomes (Figure 2C). The mineralized bone matrix was produced by OMSCs/ECs implantation in the bone environment, which may stimulate the invasion of additional osteogenic and angiogenic cells at the defect site. In this way, the co‐culture conditions, such as the cell ratio of OMSCs/ECs, may directly influence the bone regeneration.

In this study, we confirmed that the construct seeding with OMSCs/ECs mixtures at a certain ratio (0.5/1.5) promoted biomineralization and bone regeneration both in vitro and in vivo due to their synergistic effects. Further systematic studies need to illuminate the mechanism that how the vascularization of tissue‐engineering construct stimulates bone regeneration in vivo. Successful results from these studies will be beneficial in the progression of bone tissue engineering.

## 5 CONCLUSION

This study demonstrated that osteogenesis and angiogenesis could be enhanced by augmenting the paracrine effects between OMSCs and ECs interactions at an optimal ratio (0.5/1.5) in co‐culture treatment. Transplantation of an optimal ratio of OMSCs/ECs co‐culture in a scaffold, which mimics natural tissue complexities, provides a live tissue‐engineering construct that—when co‐implanted—can rapidly generate new bone tissue. The mechanism underlying this effect seems to involve the upregulation of angiogenic factors (VEGF and CD31); the key transcription factors involved in osteogenic differentiation (Msx2, Runx2 and Osterix); the subsequently increased osteogenic markers of ALP, OCN and Col‐I; and the stimulated mineralization. The findings in this study highlight that this approach holds great promise in regenerative medicine.

## CONFLICT OF INTEREST

The authors declare no competing financial interest.

## AUTHOR CONTRIBUTIONS

LM Li and JD Li conceived the study and designed the experiments. LM Li and Y Zuo designed and fabricated porous scaffold. LM Li, JD Li, Q Zou and B Cai performed in vitro and in vivo experiments. LM Li, JD Li and YB Li analysed the data and wrote the manuscript.

## Data Availability

The data that support the findings of this study are available from the corresponding author upon reasonable request.
