# Supplementary material for: Enhanced bone tissue regeneration of a biomimetic cellular scaffold with co‐cultured MSCs‐derived osteogenic and angiogenic cells
Source: Cell Prolif. 2019 Jul 11;52(5):e12658. doi: 10.1111/cpr.12658 (PMC6797511; doi:10.1111/cpr.12658)
Supplement: Supplementary file 1 [file CPR-52-e12658-s001.doc]

**1. Supplementary Figures**


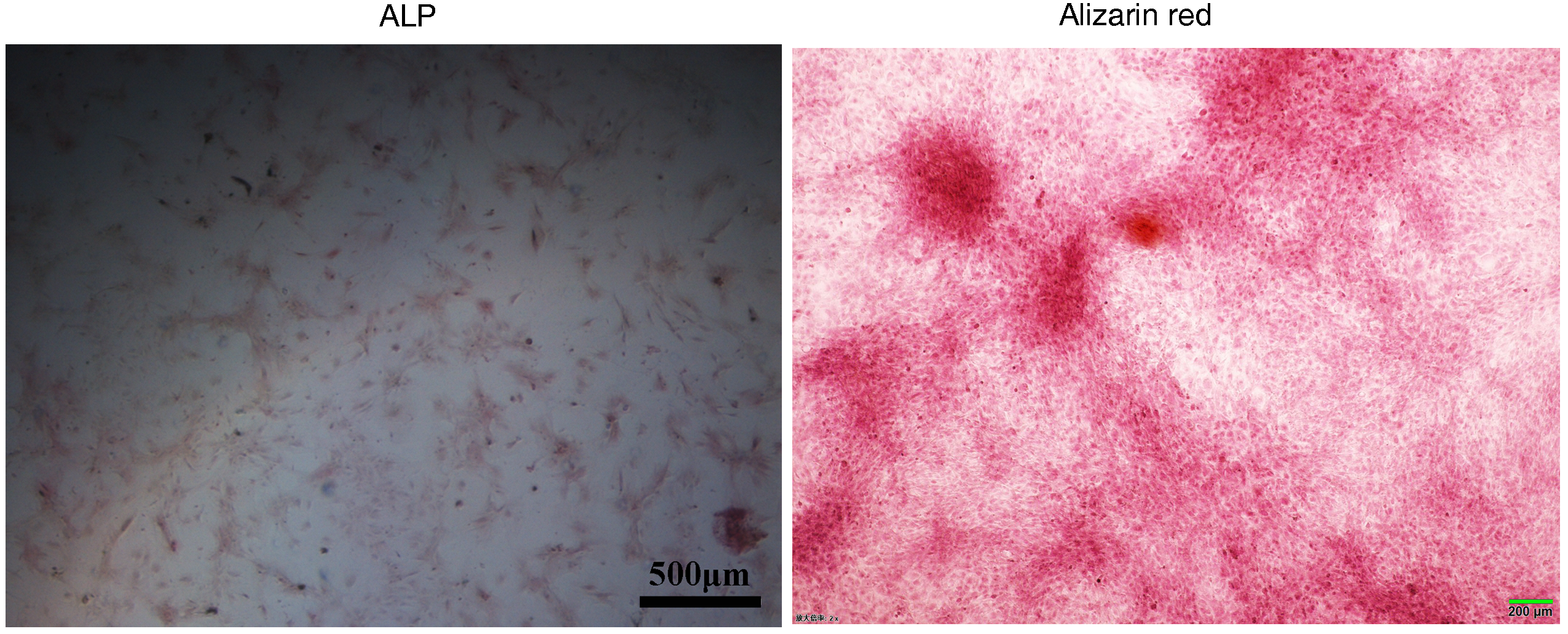


**Figure S1** Alkaline phosphatase (ALP) staining of osteogenic-differentiated cells (OMSCs) at 14 days; Alizarin red staining of OMSCs at 28 days.


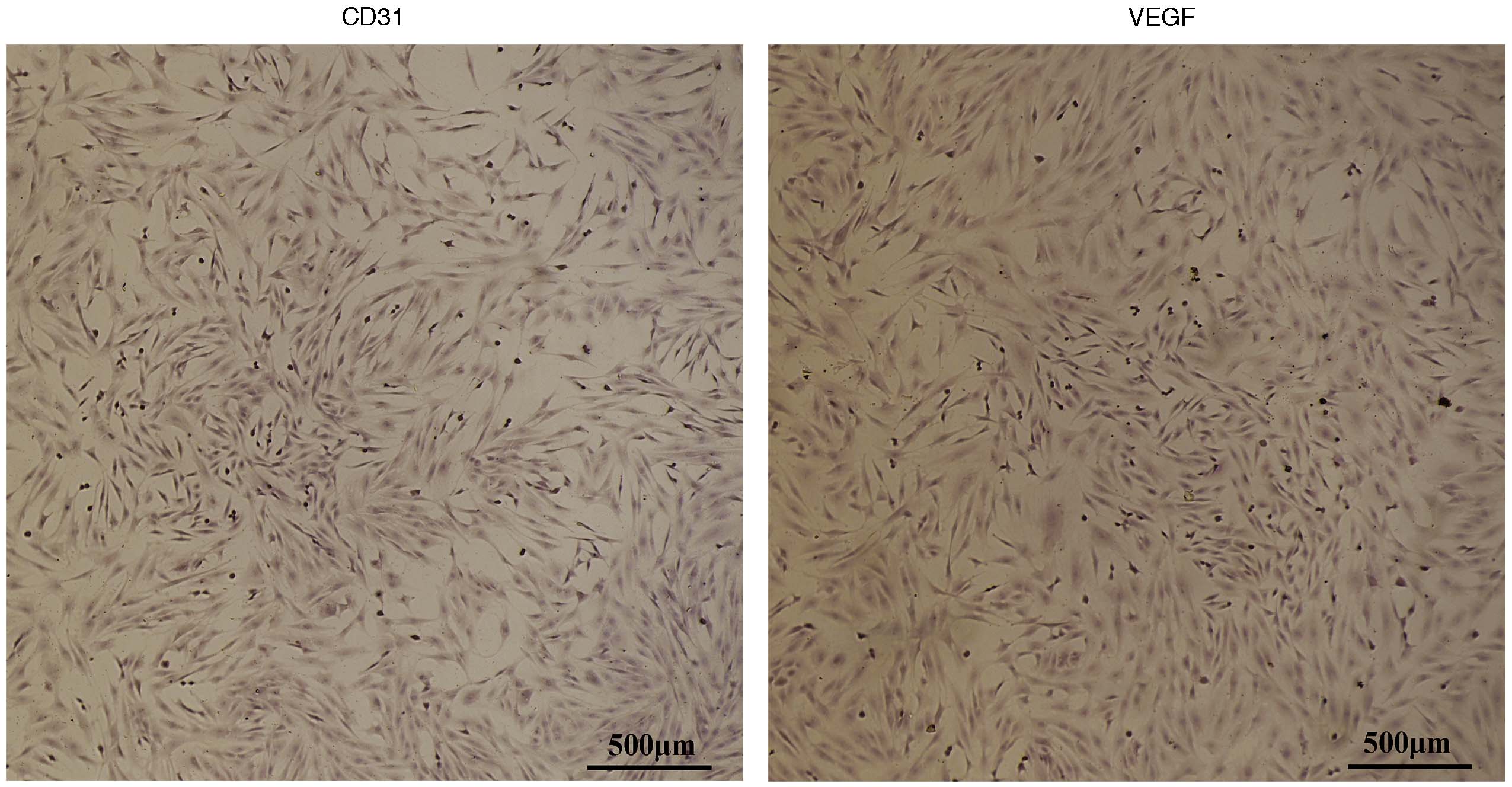


**Figure S2** CD31 staining and VEGF staining of angiogenic-differentiated cells (ECs) at 14 days.


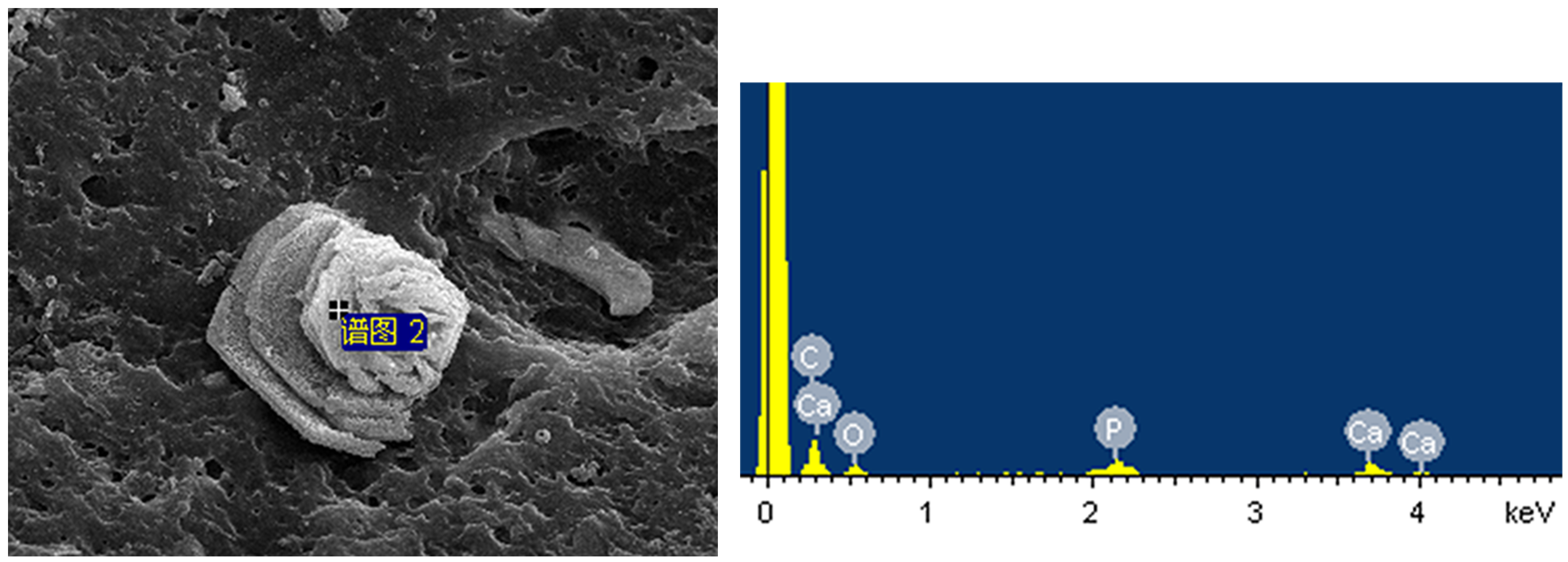


**Figure S3** SEM image of OMSCs/ECs (0.5/1.5) co-cultured after 7 days**.** EDS spectrum show that flower-like apatite contains calcium and phosphorous elements.

**2.Supplementary Table**

**Table S1** **Primer pairs used for RT-qPCR**

| Gene | NCBI gene accession number |  | Sequences |
| --- | --- | --- | --- |
| β-actin | NM_031144.3 | Forward | GCTGTGCTATGTTGCCCTAGAC |
|  |  | Reverse | CCGCTCATTGCCGATAGTGATG |
| OCN | NM_013414.1 | Forward | TGCATTCTGCCTCTCTGACC |
|  |  | Reverse | ACCACCTTACTGCCCTCCTG |
| ALP | NM_013059.1 | Forward | AACAACCTGACTGACCCTTCC |
|  |  | Reverse | CAATCCTGCCTCCTTCCAC |
| Col-I | NM_053304.1 | Forward | CCAAAGACACAGGAAATAATGC |
|  |  | Reverse | AGGTGCTGGGTAGGGAAGTA |
| VEGF | NM_001287107.1 | Forward | GTGTGGTCTTTCGTCCTTCTTA |
|  |  | Reverse | TCATCTGTCTCTCTGTCCTCTTG |
| CD31 | NM_031591.1 | Forward | GGGTGCGCCTGTAGCTAACT |
|  |  | Reverse | ATTGCTTCTCTTGACCACTTTG |
| Msx2 | NM_012982.3 | Forward | TTTGGGGTAGACAATGAAGCC |
|  |  | Reverse | TTGGGAAGAGGTGGACAGG |
| Runx2 | NM_001278483.1 | Forward | TGCCTTTAGCCCCTACACC |
|  |  | Reverse | ACATAAGTTCCCCATCTGCC |
| Osterix | NM_001037632.1 | Forward | CTATCCAGCACCCCACCTC |
|  |  | Reverse | TTGCCCACTATTGCCAACT |
